# Breakthrough in *Komagataella phaffii* cell-free protein synthesis: AOX1 promoter drives T7-independent expression efficiently: *K*. *phaffii* CFPS using a native promoter

**DOI:** 10.3724/abbs.2025115

**Published:** 2025-08-14

**Authors:** Yu Zhang, Wenjie Cong, Hualan Zhou, Jianguo Zhang

**Affiliations:** School of Health Science and Engineering University of Shanghai for Science and Technology Shanghai 200093 China

**Keywords:** cell-free protein synthesis, alcohol oxidase 1 promoter, *Komagataella phaffii*, cell extract preparation, *Pichia pastoris*

## Abstract

This study develops a cell-free protein synthesis (CFPS) system based on the endogenous alcohol oxidase 1 promoter in *Komagataella* *phaffii*. The system avoids the dependence of the T7 promoter, thus eliminating the cost issues associated with the T7 RNA polymerase-dependent system in traditional CFPS systems. By integrating an alcohol oxidase 1 promoter-driven GFP expression cassette with optimized *K*. *phaffii* cell extract, key components are optimized via a one-factor-at-a-time experiment and a deterministic screening design. This study demonstrates that potassium glutamate and magnesium glutamate have a significant synergistic effect on this system. After optimization, the system achieves a GFP yield of 596.0 mg/L, providing a new record for GFP expression in *K*. *phaffii* CFPS systems. This work provides an important theoretical foundation for the further development of T7-independent *K*. *phaffii* CFPS systems and their potential applications in scalable bioproduction.

## Introduction

Cell-free protein synthesis (CFPS) is an innovative synthetic biology technology that uses cell extracts and DNA or mRNA templates to generate proteins with the help of amino acids, enzymes, transcription factors, and nucleoside triphosphates [1]. The first CFPS system was established by Schweet *et al*. [2] in 1958 and used rabbit reticulocyte extract and ^14^C-labeled amino acids to synthesize small amounts of hemoglobin. Compared with cell-based systems, recent technological advances in CFPS enable rapid protein synthesis in hours rather than days, alongside high throughput screening capabilities and the modular assembly of synthetic biological components [ 3– 7] . Owing to the low protein production efficiency and instability of the PURE CFPS, which is composed of purified transcription and translation machinery components [ 8, 9] , the development of cell extract-based CFPS systems has attracted the attention of researchers and industrial communities, such as rabbit reticulocytes [10], wheat germ [11], insect cells, *E*. *coli*, and yeast cell [12] extract-based CFPS systems.

Currently, *E*. *coli* CFPS dominates all the other CFPS systems because of its available components and well-established protocol of protein synthesis. *E*. *coli* cell extract-based CFPS also performs well in the biosynthesis of functional compounds containing chloro, alkene, and alkyne groups from unnatural amino acids [13], screening and identifying new enzymes from various species [14]. Additionally, *E*. *coli* cell extract-based CFPS was harnessed to investigate the interactions between proteins [15] and peptides with post-translation modification [16]. *E*. *coli* cell extract-based CFPS uses the T7 promoter and optimized T7 RNA polymerase [17] and works on a 10 mL scale successfully [18]. However, heterologous protein expression in *E*. *coli* often faces the issue of a lack of post-translational modifications, which can lead to misfolding, insolubility, or functional loss of heterologous proteins [19]. In recent years, other prokaryotic CFPS have also shown remarkable performance. Shin *et al*. [20] used the holoenzyme E(70) bacteriophage T7 to express 60 genes in a 40 kbp DNA fragment. Moore *et al*. [21] developed *Streptomyces venezuelae* CFPS via the Streptomyces kasOp* promoter and further demonstrated protein synthesis from high-GC-content genes [22]. Xu *et al*. [23] also reported *Streptomyces* cell extract-based CFPS after screening native promoters and ribosome binding sites, with a final yield of 515.7 ± 25.3 mg/L green fluorescent protein (GFP). However, prokaryotic CFPs generally lack eukaryotic post-translational modifications (PTMs), limiting their utility for complex proteins. The common issue of low reporter expression when the native promoter is used is the low level of RNA polymerase, which is crucial for CFPS systems [24]. Therefore, CFPS with high RNA polymerase activity and efficient promoters from eukaryotic organisms has become a promising option for heterologous protein expression. Gupta *et al*. [25] used the tobacco *Nicotiana tabacum* BY‐2 cell extract CFPS, which was commercialized as ALiCE®, to express diverse, functional proteins at high yields of 1.5 mg/mL in 48 h.

The methylotrophic yeast *Komagataella phaffii*, formerly known as *Pichia pastoris*, has been a famous cell factory for heterologous protein production for years [26]. It has also been used as a source of cell extract for *K*. *phaffii* CFPS by several researchers [27]. On the basis of the well-established protocol of *E*. *coli*-based CFPS, the *K*. *phaffii* CFPS system relies on optimized extract preparation and compatible T7 promoter-driven templates in *E*. *coli* CFPS [ 28, 29] . Zhang *et al*. [30] simplified CFPS cell extract preparation and reaction conditions, achieving 50.16 ± 7.49 mg/L GFP in a 5 h batch reaction. Spice *et al*. [31] also developed a *K*. *phaffii*-based CFPS after reaction composition optimization through minimized experimental design and demonstrated the capacity of human serum albumin production. The internal ribosome entry site (IRES) and Kozak sequences used in the *K*. *phaffii* CFPS assembly protocol were validated by Aw *et al*. and Spice *et al*. [ 32– 34] . For example, Wang *et al*. [35] investigated the influence of IRES and Kozak sequences and obtained a *K*. *phaffii* CFPS after optimization of a cell extract, energy regeneration system (creatine phosphate and creatine phosphate kinase), and metal ions (K ^+^ and Mg ^2+^). Although *K*. *phaffii*, as a microbial cell host, is widely used for the expressions of more than 5000 proteins, the application of its CFPS is still limited by the reliance on the expensive T7 RNA polymerase to drive gene transcription via the T7 promoter. As a result, *K*. *phaffii* CFPS has been reported in only a few cases. Currently, the optimization of other components in the *K*. *phaffii* CFPS system has not yielded any significant improvements, although most researchers indicate that reduced costs remain the primary driving force for *K*. *phaffii* CFPS development [36]. In addition, systematic replacement of T7-dependent transcription or optimization of endogenous mechanisms, such as natural promoters or RNA polymerases, remains underexplored, which highlights a key direction for future research.

Many researchers have emphasized the application of native promoters in the production of intracellular recombinant proteins by *K*. *phaffii* [37]. The *K*. *phaffii* alcohol oxidase 1 promoter ( *P _AOX1_*) is one of the most powerful promoters of gene expression [ 38, 39] . In native *K*. *phaffii* cells, alcohol oxidase 1 mRNA driven by *P _AOX1_* contributed 5% of the total mRNA in *K*. *phaffii* cells during the methanol induction phase. Alcohol oxidase 1 accounted for 30% of the total protein in *K*. *phaffii* cells. Heterologous genes under *P _AOX1_* have been widely applied to reach high expression levels [40]. Therefore, utilizing *P _AOX1_* in the *K*. *phaffii* CFPS can eliminate the dependence on T7 RNA polymerase and fully unleash the significant potential of *K*. *phaffii* for protein synthesis in the CFPS.

## Materials and Methods

### Strains, vectors, chemicals, and medium

The strains *K*. *phaffii* GS115 and its protease-deficient derivative SMD1163 lack proteinase A and proteinase B [41]. *E*. *coli* DH5α and the vectors pPIC9k (Thermo Fisher Scientific, Massachusetts, USA) and pET25b (Merck, Darmstadt, Germany) were used in this study. All primers listed in Supplementary Table S1 were synthesized by Sangon Biotech (Shanghai, China). The cells were cultured in YPD (1% yeast extract, 2% tryptone, and 2% glucose), BMGY (1% yeast extract, 2% tryptone, 1% glycerol, 1.34% YNB, and 4 × 10 ^–5^% biotin, pH 6.0), minimal dextrose medium (1.34% YNB, 2% glucose, and 4 × 10 ^–5^% biotin), or LB medium (0.5% yeast extract, 1% tryptone, and 1% NaCl) supplemented with appropriate antibiotics (LB: 100 μg/mL ampicillin and 50 μg/mL kanamycin; YPD: 0.25 mg/mL G418 and 0.1 mg/mL zeocin). All chemicals were obtained from Sinopharm Chemical Reagent (Shanghai, China) and all biochemicals were from Sangon Biotech.

### Strain construction

The construction process of the recombinant vector is shown in Supplementary Figure S1. The *IKEP* gene fragment, which includes the cricket paralysis virus IRES, Kozak sequence, *GFP* gene, and polyA, was synthesized by GenScript Biotech Corporation (Nanjing, China) and amplified via the primers IKEP-F and IKEP-R. Next, the pPIC9K plasmid was double-digested with *Bam*H I and *Not* I to remove the PAOX1-AOX1 T internal fragment, followed by the fusion of the IKEP fragment via In-Fusion technology to construct the recombinant plasmid 9kI. The 9kI plasmid was linearized with *Sal* I and then electroporated into *K*. *phaffii* GS115 cells according to the method described previously [42]. Transformants were selected on MD agar plates containing 0.1% G418, and positive clones were validated via PCR via the primers IKEP-F and IKEP-R. Subsequently, the AIA fragment containing *P _AOX1_* and AOX1 T was amplified from the recombinant 9kI plasmid (using the primers AIA-F and AIA-R) and inserted into the pET25b-ΔlacI vector to construct the ΔpAIA plasmid for *K*. *phaffii* CFPS experiments. Simultaneously, the IKEP fragment was fused with the pET25b-ΔlacI vector to construct the negative control plasmid ΔpI for *K*. *phaffii* CFPS experiments. Routine molecular operations were carried out according to the molecular cloning protocol [43]. Both plasmids were amplified in *E*. *coli* BL21. All the recombinant vectors and strains were confirmed via DNA sequencing at the Beijing Genomics Institute (Beijing, China).

### Recombinant *K*. *phaffii* 9kI cultivation for GFP expression

Recombinant *K*. *phaffii* 9kI was cultivated in 50 mL of YPD medium in a 250-mL flask at 30°C and 200 rpm for 18–24 h until it reached an OD _600_ value of approximately 2.0–8.0. The cells were subsequently transferred into 25 mL of BMGY medium in 50-mL tubes at a 4% inoculum ratio and incubated at 200 rpm for 60 h to exhaust glycerol. Then, methanol was added every 24 h at a final concentration of 1% to induce GFP expression. *K*. *phaffii* GS115 was also cultivated simultaneously as a control.

### *K*. *phaffii* cell extract preparation

*K*. *phaffii* SMD1163 was transferred from a stock tube into an YPD plate for cultivation at 30°C, and one colony was picked into 100 mL of YPD in a 250-mL flask for overnight cultivation at 30°C and 200 rpm. The cells were subsequently transferred to four 1-L flasks, each containing 250 mL of BMGY with a 4% inoculum ratio, resulting in an initial OD _600_ of 0.05. The flasks were incubated at 30°C with shaking at 200 rpm for 60 h. Methanol (1%) was added every 24 h to induce *P _AOX1_* transcription. After 72 h of induction, the cells were harvested via centrifugation at 8000 *g* for 15 min at 4°C.

Two approaches, S60 and S301, were used to prepare *K*. *phaffii* cell extracts in this study. *K*. *phaffii* cell extract was prepared via the S60 approach, adapted from Hodgman *et al*. [44]. The collected wet cell pellets were washed with cold washing buffer (30 mM HEPES pH 7.4, 100 mM potassium acetate, 2 mM magnesium acetate, 2 mM dithiothreitol, and 8.5% W/V mannitol) at 4°C. The cells were resuspended and centrifuged three times. The pellets were mixed with cold lysis solution (same composition plus 0.5 mM PMSF) and sonicated for 60 min via a JL-650 W ultrasonic probe (Ф6 mm; Jinlan, Shanghai, China) with 1 s intervals and 1.5 s pulses at 20 W. After two rounds of centrifugation at 30,000 *g* (CR21N; Hitachi, Tokyo, Japan) for 30 min at 4°C, the supernatant was collected and dialyzed at 4°C via a 3.5-kDa dialysis tube. Following four rounds of dialysis, the extract was further centrifuged twice at 21,000 *g* for 30 min to obtain the final cell extract for CFPS.

The S301 approach was an improvement over the S30 method [45], with cell lysis performed via the S60 method, followed by centrifugation at 30,000 *g* for 30 min twice to obtain the supernatant. This mixture was mixed with a preculture mixture (25 mM HEPES-KOH pH 7.4, 12 mM magnesium glutamate, 0.415 mM NTP, 0.1 mM mixed amino acids, 50 mM creatine phosphate, and 1.7 mM dithiothreitol) at a 10:3 V/V ratio and incubated at 30°C for 80 min with shaking in a 100 rpm shaker (THZ-312; Jinghong, Shanghai, China). The mixture was dialyzed at 4°C using a 3.5-kDa dialysis tube and cold lysis solution (100× final volume). After four cycles of dialysis, the extract was centrifuged twice at 4,000 *g* for 30 min to obtain the final cell extract for CFPS.

### *K*. *phaffii* CFPS set up

The *K*. *phaffii* CFPS reaction mixture (15 μL) contained 25 mM HEPES-KOH (pH 7.4), 60 mM potassium glutamate, 9.0 mM magnesium glutamate, 0.4 mM ATP, GTP, CTP, and UTP; 0.1 mM 20 amino acids; 40 mM creatine phosphate; 1.7 mM DTT; 1.0 mM putrescine; 0.5 mM spermidine; 0.27 mg/mL creatine phosphate kinase (from rabbit muscle, Sigma-Aldrich, St Louis, USA); 16.7 μg/mL ΔpAIA for GFP expression (or ΔpI as a control); and 7.5 μL *K*. *phaffii* cell extract. The reaction mixtures were incubated at 24°C for 5 h. Amino acids solution were prepared in advance and stored at –80°C.

### *K*. *phaffii* CFPS optimization

On the basis of our previous optimization of *K*. *phaffii* CFPS via the T7 promoter [46], seven components (HEPES, NTP, amino acids, creatine phosphate, plasmids, potassium glutamate and magnesium glutamate) were optimized via a definitive screening design (DSD). Four key components (potassium glutamate, magnesium glutamate, NTP and creatine phosphate) were optimized first by one-factor-at-a-time experiments, followed by DSD. The experimental design (4 factors, 3 levels) was generated via JMP (SAS Institute Inc., Cary, USA). Model significance was tested via analysis of variance (ANOVA), and the standard deviation was used to evaluate the model’s accuracy.

### Fluorescence intensity determination

After the *K*. *phaffii* CFPS reaction, 2 μL of the CFPS solution was diluted 50-fold with deionized water. The fluorescence intensity was measured via a multifunctional spectrometer (SpectraMax M2; Shanghai Shanpu Biotechnology Co., Ltd., Shanghai, China) with excitation at 488 nm and emission at 510 nm. The vector ΔpI was used as a control, and the fluorescence intensity was calculated by subtracting the control values. The GFP yield was determined via a standard curve of GFP (C600323; Sangon Biotech) concentration vs fluorescence.

### Western blot analysis of GFP

*K*. *phaffii* CFPS samples were mixed with loading buffer, boiled for 10 min, and loaded onto a 15% SDS-PAGE gel (PG114; Shanghai Epizyme Biomedical Technology Co., Ltd., Shanghai, China). The electrophoresis was run at 100 V for 120 min in Tris-glycine buffer (24.8 mM Tris base, 191.8 mM glycine, 3.5 mM SDS). The PVDF membrane (Amresco Inc, Solon, USA) was activated with methanol for 10 s, washed with water, and soaked in transfer buffer (24.8 mM Tris base, 191.8 mM glycine, 20% methanol) for 10 min. Protein transfer was carried out at 100 V for 90 min in a cold environment. Western blotting was performed using a GFP-tagged protein detection kit (P0986S; Beyotime, Shanghai, China), and the results were visualized with a ChemiDoc MP system (Bio-Rad, Hercules, USA).

### Miscellaneous determination

The cell density was determined by measuring the absorbance at 600 nm via a SpectraMax M2 spectrometer (Shanghai Shanpu Biotechnology Co., Ltd.). SDS-PAGE was performed following the molecular cloning protocol [43]. The protein content was determined via the Bradford method [47] using coomassie brilliant blue R250.

### Statistical analysis

All experiments in this study were carried out in triplicate. The standard deviation was calculated using Microsoft Excel 2016 (Microsoft, Seattle, USA). Graphs were created using GraphPad Prism (GraphPad Software, La Jolla, USA). Response surface analysis was performed using JMP (Johns Hopkins University/Université de Sciences et de Technologie de Lille). Statistical significance was determined using appropriate statistical tests ( *e.g*., *t*-tests or ANOVA), with a significance level set at *P*  < 0.05.

## Results

### GFP expression by the recombinant *K*. *phaffii* 9KI strain

**Figure 1:**
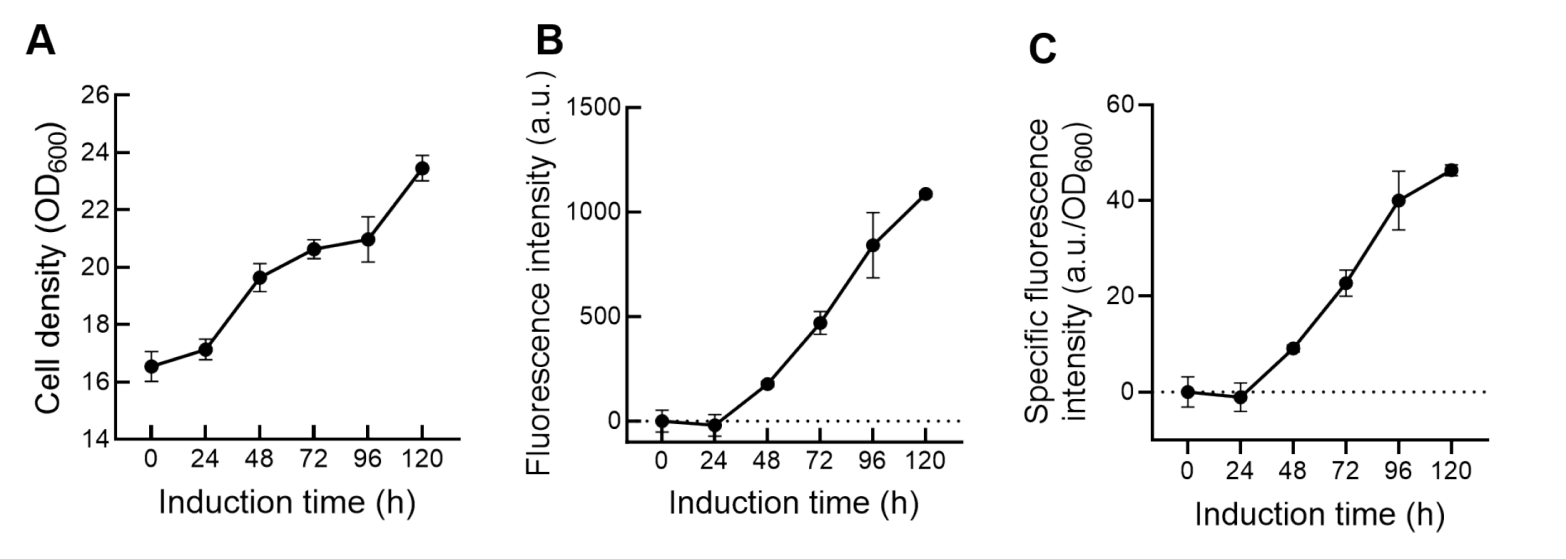
Figure 1 GFP expression by *K*. *phaffii* 9KI The data shown are from a single representative experiment out of three replicates. (A) Cell density changes of K. phaffii 9KI during methanol induction after 60 h cultivation in BMGY medium containing glycerol. (B) GFP fluorescence intensity of K. phaffii 9KI during methanol induction in BMGY medium. (C) Specific fluorescence intensity of K. phaffii 9KI during methanol induction in BMGY medium.

To confirm the functional fragment AIA, recombinant *K*. *phaffii* 9KI was cultivated with glycerol and induced with 1% methanol to express GFP, the results of which are shown in Figure 1. After 60 h of cultivation of *K*. *phaffii* 9KI in BMGY containing glycerol, the cell density of *K*. *phaffii* 9KI increased from 16.54 OD _600_ to 23.56 OD _600_ during the subsequent 120 h of methanol induction ( Figure 1A). The fluorescence intensity of *K*. *phaffii* 9KI steadily increased to 1087.18 a.u. after 120 h, with no fluorescence observed in the first 24 h ( Figure 1B). The specific fluorescence intensity reached 46.36 a.u./OD _600_ ( Figure 1C). Therefore, the AIA fragment was then circularized into the pET25b plasmid and used for *K*. *phaffii* CFPS.

### Influence of the *K*. *phaffii* cell extract preparation on the CFPS

**Figure 2:**
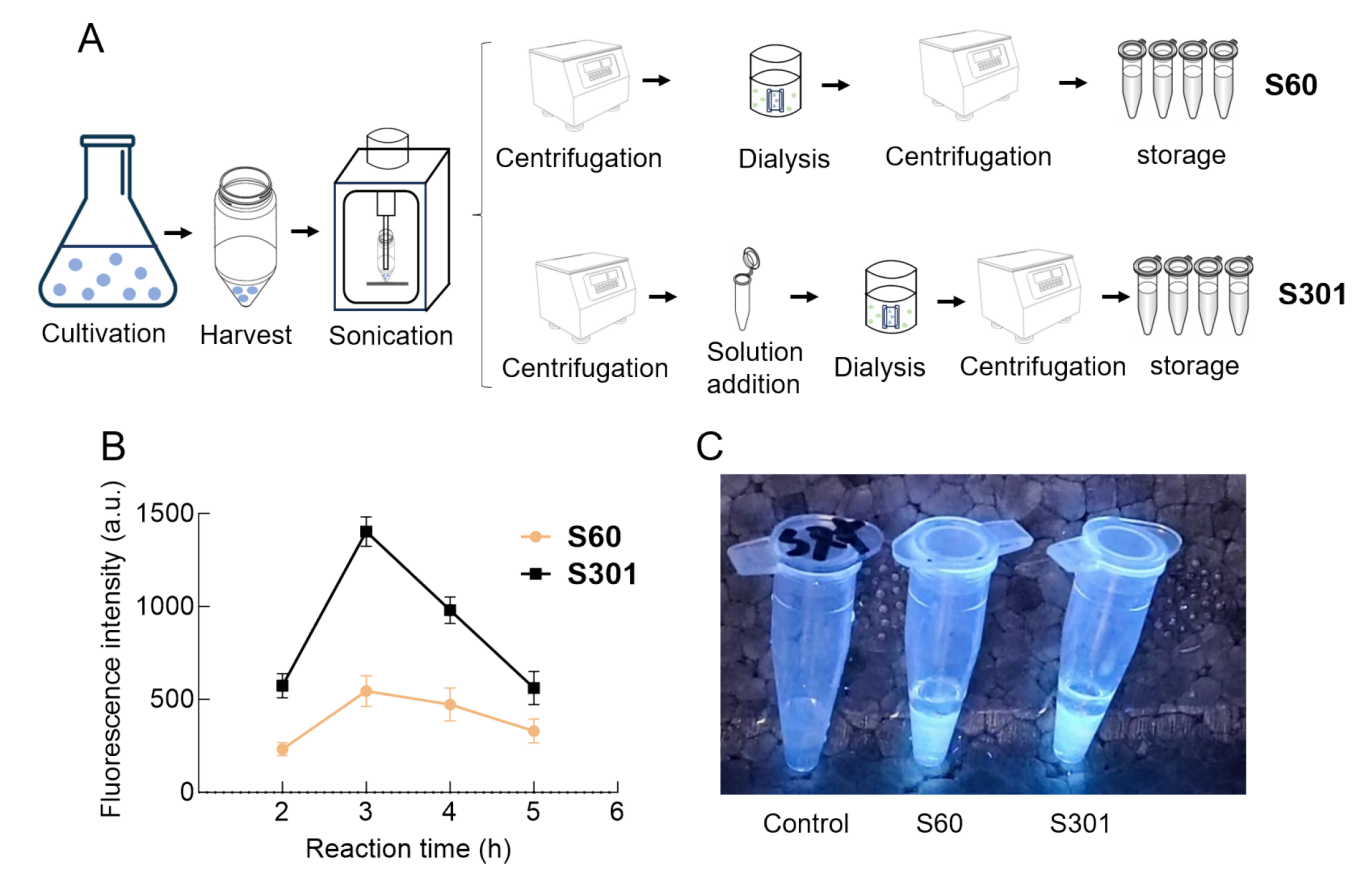
Figure 2 Influences of two procedures of *K*. *phaffii* cell extract on GFP expression (A) Two procedures of K.phaffii cell extract, S60 and S301. (B) Fluorescence intensity curve of cell extracts prepared using the S60 and S301 methods in K. phaffii CFPS after 5 h of reaction. The data, which have been corrected for the control, are from one representative experiment out of three replicates. (C) Fluorescence photographs of K.phaffii CFPS using S60 and S301.

*K*. *phaffii* SMD1163 was cultured in BMGY medium with glycerol as the sole carbon source. After glycerol exhaustion at 60 h, methanol was added to induce *P _AOX1_* transcription. The methanol-induced growth curves are shown in Supplementary Figure S2. S60 is the commonly used lysate preparation method for *K*. *phaffii* CFPS, where the centrifugation time is 30 min, which is repeated twice, for a total of 60 min. Therefore, it is abbreviated as S60. S301 is a novel lysate that we developed by combining the S30 method with the traditional *K*. *phaffii* lysate preparation (S60) and an incubation process. Hence, it is abbreviated as S301. Figure 2A shows the differences between the two preparation methods of *K*. *phaffii* cell extract. The fluorescence intensities of *K*. *phaffii*-based CFPS using S60 and S301 showed mountain-like curves within 5 h of reaction ( Figure 2B) and reached the highest value of 545.53 a.u. and 1403.03 a.u. after 3 h of reaction, respectively. Additionally, *K*. *phaffii* CFPS systems at 3 h are shown in Figure 2C, in which the vector ΔpI was used in the control group. We also determined the protein content of S301 to be 943.2 ± 20 mg/L, which was 6.1-fold greater than that of the cell extract produced by glass bead shaking (153.8 ± 10 mg/L) [48]. The standard curve of the GFP fluorescence intensity versus the GFP yield is shown in Supplementary Figure S3. The *K*. *phaffii* CFPS constructed in this study achieved the highest GFP yield at 3 h, which was significantly greater than that of other yeasts’ CFPS [ 30, 44] . The ΔpI vector without *P _AOX1_* did not produce fluorescence, confirming the necessity of *P _AOX1_* for GFP synthesis. Additionally, GFP expression was unsuccessful without the IRES and Kozak sequences, which are required for efficient translation.

### CFPS optimization through one-factor-at-a-time experiment

**Figure 3:**
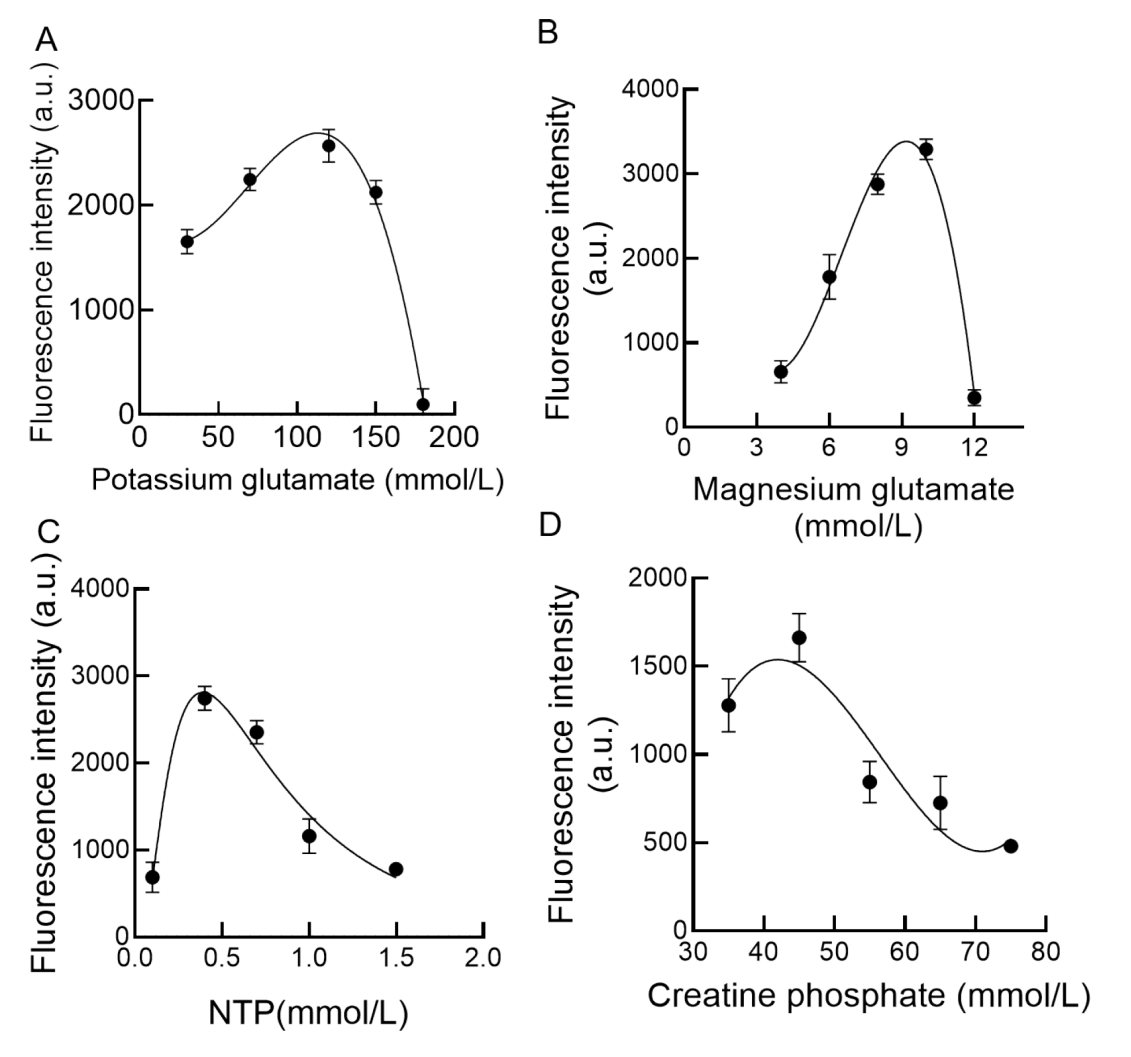
Figure 3 Optimization of *K*. *phaffii* CFPS by one factor at a time The data, corrected for the control, are from one representative experiment out of three replicates. (A) Fluorescence values of different concentrations of potassium glutamate in K.phaffii CFPS after 3 h of reaction. (B) Fluorescence values of different concentrations of magnesium glutamate in K.phaffii CFPS after 3 h of reaction. (C) Fluorescence values of different concentrations of NTP in K.phaffii CFPS after 3 h of reaction. (D) Fluorescence values of different concentrations of creatine phosphate in K.phaffii CFPS after 3 h of reaction.

On the basis of our previous study on *K*. *phaffii* CFPS utilizing the T7 promoter and T7 RNA polymerase, four key components significantly influence protein synthesis. Therefore, this study optimized these four critical components: magnesium ions, phosphocreatine, potassium glutamate, and magnesium glutamate. Magnesium ions facilitate tRNA aminoacylation and ribosome stabilization, whereas creatine phosphate supports ATP regeneration. The highest GFP fluorescence intensities were 1954.41 a.u., 2471.88 a.u., 2058.94 a.u., and 1257.65 a.u. at 70–150 mM potassium glutamate, 4–8 mM magnesium glutamate, 0.7–1.5 mM NTP, and 35–55 mM creatine phosphate, respectively, as shown in Figure 3. These findings highlight the need for response surface optimization to maximize CFPS performance.

### DSD optimization of the CFPS

**Figure 4:**
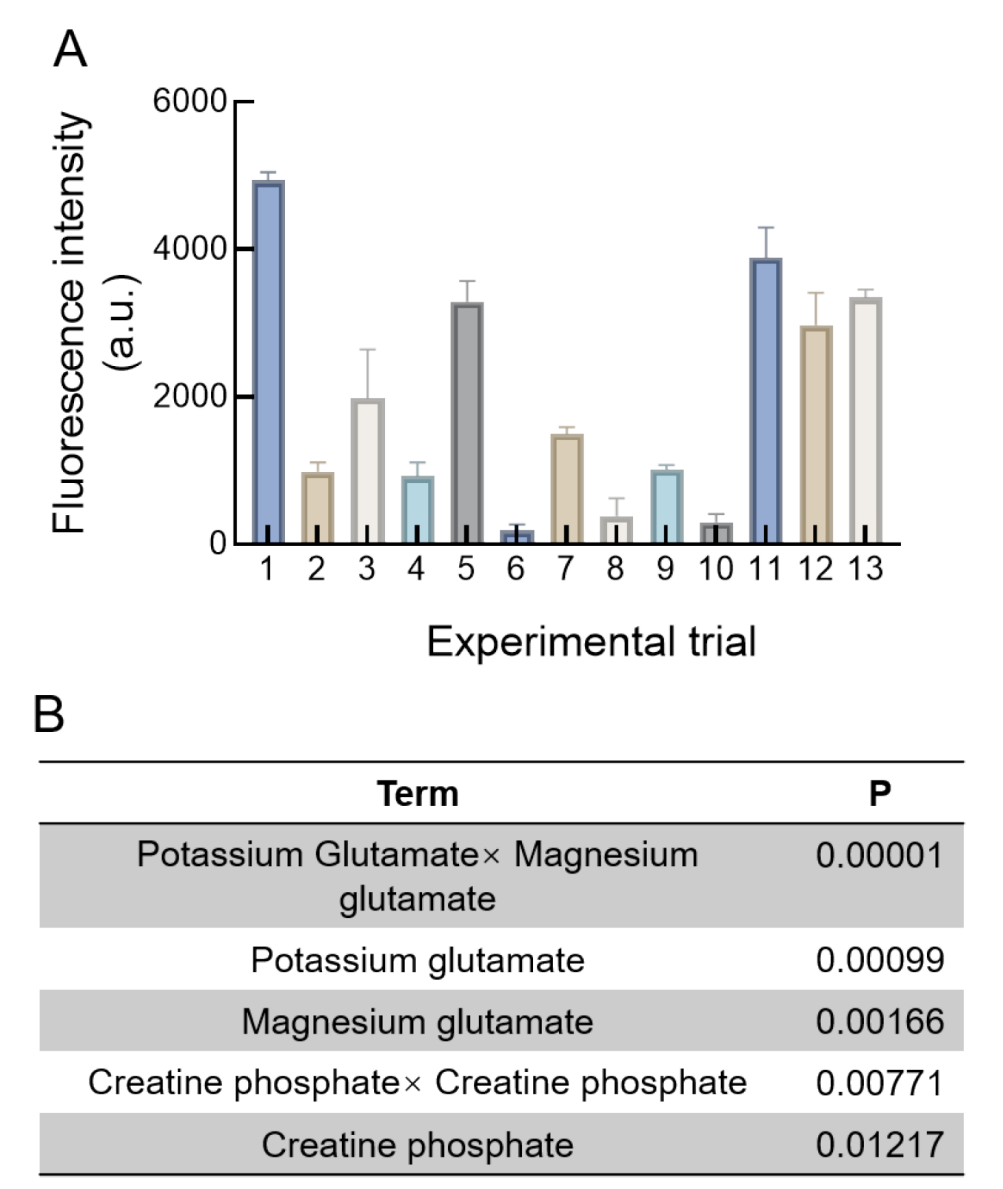
Figure 4 Fluorescence intensities of *K*. *phaffii* CFPS in DSD optimization (A) Fluorescence intensity of 13 K.phaffii CFPS systems at 3 h, analyzed using the DSD approach and JMP software. The data, corrected for the control, are from one representative experiment out of three replicates. (B) Important factors and significant interaction factors identified in the DSD model that affect K. phaffii CFPS, with P < 0.05.

The fluorescence intensities of the 13 *K*. *phaffii* CFPS systems at 3 h shown in Figure 4A were analyzed via the DSD approach shown in Supplementary Table S2 and JMP software. All fluorescence intensities fell into a range between 185.69 a.u. and 4936.08 a.u. and were fitted with a first-order polynomial model ( Equation 1). The *P* values of the five terms in Equation 1 were significant ( Figure 4B). NTP did not significantly influence the fluorescence intensity.

GFP(a.u.)=1275.79+350.76×(A−4510)−591.48×(B−11040)+534.73×(C−102)+(A−4510)×((A−45)×915.0610)+(D−11040)×((C−10)×(−1772.55)2)Equation 1

A: mmol creatine phosphate; B: mmol potassium glutamate; C: mmol magnesium glutamate; D: mmol potassium glutamate.

The fitness of model was validated through the positive correlation relationship between predicted fluorescence intensity and the measured fluorescence intensity with several regression coefficients: RMSE 298.31, R ^2^ = 0.98, and *P*  < 0.0001. Interactions among factors were observed between potassium glutamate and magnesium glutamate only. The fluorescence intensity was estimated to be in the range of 4880.59~6000.18 a.u. ( Supplementary Figure S4). Creatine phosphate, potassium glutamate, and magnesium glutamate influenced the fluorescence intensities, as shown by the different profiles in Supplementary Figure S4. Optimization of the system resulted in a fluorescence intensity of 4950.34 a.u. at 55 mM creatine phosphate, 70 mM potassium glutamate, and 12 mM magnesium glutamate.

**Figure 5:**
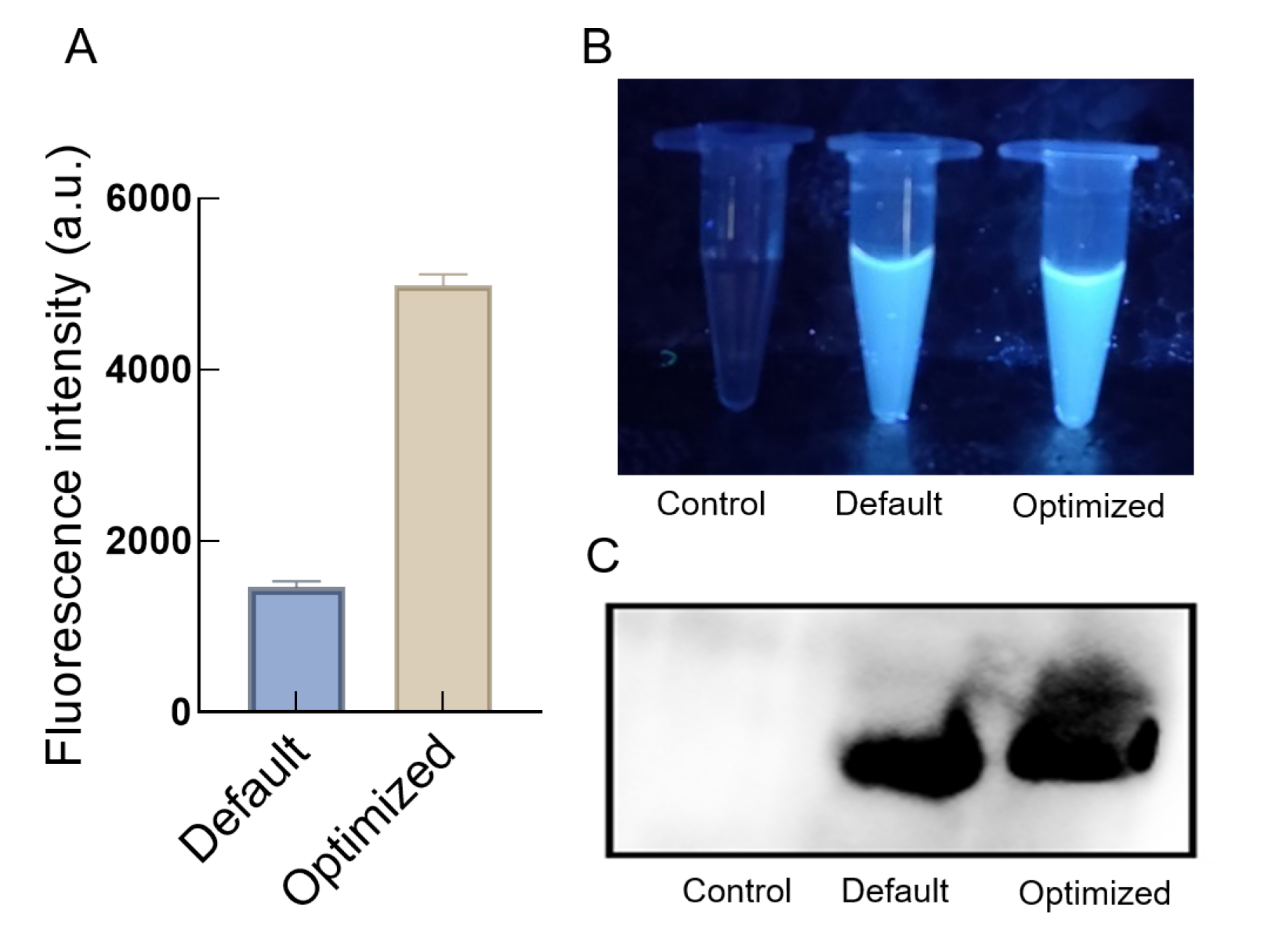
Figure 5 Confirmation of fluorescence intensities of GFP in *K*. *phaffii* CFPS after 3 h of reaction through DSD optimization “Default” refers to the preoptimization system and “Optimized” refers to the postoptimization system. (A) Comparison of fluorescence values of K.phaffii CFPS before and after optimization. The data, corrected for the control, are from one representative experiment out of three replicates. (B) Photographs. (C) Western blot of GFP.

The optimized *K*. *phaffii* CFPS showed 4984.51 a.u. GFP fluorescence intensity ( Figure 5A), matching the predicted values. A GFP yield of 596.0 mg/L was also achieved a 3.4-fold increase over that of other *K*. *phaffii* CFPS. The final GFP samples with fluorescence of *K*. *phaffii* CFPS are shown in Figure 5B. Western blot analysis confirmed that GFP was expressed ( Figure 5C).

## Discussion

In this study, we developed a novel *K*. *phaffii* CFPS system by engineering *P _AOX1_* and optimizing a cell extract preparation named S301, which achieved 2.6-fold greater fluorescence intensity than conventional methods. Furthermore, this *K*. *phaffii* CFPS was optimized to obtain a reaction system containing one factor at a time, and DSD optimization was performed with a further 3.4-fold increase in GFP expression. In the S301 system, magnesium glutamate and creatine phosphate were replaced by magnesium acetate and potassium acetate in the pre-culture solution. This change was made because the electron charge density of glutamate and phosphate is more evenly distributed compared to acetate, which significantly improved the stability and dispersion of the solution [ 49, 50] . Additionally, the use of ultrasonic cell disruption and the addition of mannitol in the cold lysis buffer helped maintain the stability of the proteins in the lysate [51]. The design of this system successfully avoids the use of costly T7 RNA polymerase, employing native *P _AOX1_* for transcription, thereby significantly reducing production costs ( Table 1). T7 RNA polymerase typically accounts for 65% of the total CFPS cost [ 52, 53] . This T7-independent platform not only achieved the highest GFP expression levels reported in *K. phaffii* CFPS but also laid a solid foundation for future efforts to further enhance protein synthesis efficiency and maintain cost-effectiveness through native and engineered promoters.

**Table:** **Table 1** Summary of T7 RNA polymerase used in current *K*. *phaffii* CFPS systems

CFPS	Promoter	Amount of RNA polymerase	Expression level	Time (h)	Ref.
*K*. *phaffii*	*T*7	4 U/μL RNA polymerase	50.16 mg/L GFP	5	[31]
*K*. *phaffii*	*T*7	4 U/μL RNA polymerase	100.0 mg/L Human serum albumin	7	[32]
*K*. *phaffii*	*T*7	4 U/μL RNA polymerase	116.0 mg/L Human serum albumin	4	[33]
*K*. *phaffii*	*T*7	No data RNA polymerase	300.0 mg/L GFP	6	[35]
*K*. *phaffii*	PAOX1	/	596.0 mg/L GFP	3	This study

In conclusion, this study provides an efficient, cost-effective, and high-yield *K*. *phaffii* CFPS platform that offers strong support for the expansion of eukaryotic CFPS applications. Future research will focus on enhancing translation efficiency and optimizing chaperone systems to facilitate the production of complex proteins.

## Supporting information

Supplementary_materials_C1

## Supplementary Data

Supplementary data is available at *Acta Biochimica et Biophysica Sinica* online.
