# Supplementary material for: Breakthrough in Komagataella phaffii cell-free protein synthesis: AOX1 promoter drives T7-independent expression efficiently : K. phaffii CFPS using a native promoter
Source: Acta Biochim Biophys Sin (Shanghai). 2025 Aug 14;58(3):691–9. doi: 10.3724/abbs.2025115 (PMC13059779; doi:10.3724/abbs.2025115)
Supplement: Supplementary_materials_C1 [file Supplementary_materials_C1.docx]

**Supplementary Table S1. Primers used in this study**

| Primers | Sequence（5′→3′） | Function |
| --- | --- | --- |
| IKEP-F | CAACTAATTATTCGAAGGATCCCGAATCTAGAAAGCAAAAATGTGATCTTGC | IKEP amplification |
| IKEP-R | AGGCGAATTAATTCGCGGCCGCGTGGTGGTGGTGGTGGTGC | IKEP amplification |
| AIA-F | AGTGCGGCGACGATAGTCGATCTAACATCCAAAGACGAAAGGTTGAATGA | AIA amplification |
| AIA-R | CATTCTGACATCCTCTTGAGCTCAGCGGTGGCAG | AIA amplification |
| ΔpET25b-F | CTGAAAGGAGGAACTATATCCGGATTGG | pET25b-Δlac I |
| ΔpET25b-R | GACTATCGTCGCCGCAC | pET25b-Δlac I |
| con-IKEP-F | AAGTGCGGCGACGATAGTCTCTAGAAAGCAAAAATGTGATCTTGCTTGTAAATAC | IKEP as control |
| con-IKEP-R | TATAGTTCCTCCTTTCAGGCTCAGCGGTGGCAG | IKEP as control |

**Supplementary Table S2.** **Selected parameters and their vales for *K. phaffii* CFPS during** **DSD optimization**

| No. | Potassium glutamate (mmol/L) | Magnesium glutamate (mmol/L) | NTP (mmol/L) | Creatine phosphate (mmol/L) |  |
| --- | --- | --- | --- | --- | --- |
| 1 | 150 | 8 | 1.5 | 35 |  |
| 2 | 110 | 4 | 1.5 | 35 |  |
| 3 | 110 | 8 | 0.7 | 55 |  |
| 4 | 150 | 6 | 0.7 | 35 |  |
| 5 | 70 | 4 | 0.7 | 55 |  |
| 6 | 110 | 6 | 1.1 | 45 |  |
| 7 | 70 | 6 | 1.5 | 55 |  |
| 8 | 70 | 4 | 1.1 | 35 |  |
| 9 | 150 | 4 | 1.5 | 55 |  |
| 10 | | 70 | 8 | 0.7 | 35 |
| 11 | | 150 | 8 | 1.1 | 55 |
| 12 | | 70 | 8 | 1.5 | 45 |
| 13 | | 150 | 4 | 0.7 | 45 |

**Supplementary Figure S1.** **The overall process of recombinant vector construction for *K. phaffii* CFPS** (A) Construction process of vector 9KI. (B) Construction process of vector ΔpAIA. (C) Construction process of vector ΔpI.

**Supplementary Figure S2.** **Growth curves of *K. phaffii* strains**

**Supplementary Figure S3**. **Standard curve of GFP fluorescence versus GFP yield**

**Supplementary Figure S4**. **Analysis of DSD optimization** (A) Relationship between predicted fluorescence intensity and experimental results. (B) Interactions between different factors. (C) Prediction profiler of DSD model.
